# The effects of foods available through the Food Distribution Program on Indian Reservations (FDPIR) on inflammation response, appetite and energy intake

**DOI:** 10.1017/S1368980020002852

**Published:** 2020-09-01

**Authors:** Melinda Smith, Elizabeth Rink, Suzanne Held, Carmen Byker Shanks, Mary P Miles

**Affiliations:** 1 Nutrition Research Laboratory, Department of Health and Human Development, Montana State University, PO Box 173540, Bozeman, MT 59717, USA; 2 Montana State University, Bozeman, MT, USA

**Keywords:** Food Distribution Program on Indian Reservations, Commodities, Inflammation, Energy intake, Appetite

## Abstract

**Objective:**

To compare the effects of a typical Food Distribution Program on Indian Reservations (FDPIR) diet with an FDPIR diet that meets Dietary Guidelines for Americans (DGA) on inflammation response, appetite and energy intake on a combination of American Indian (AI) and non-AI individuals.

**Design:**

A within-subjects, randomised, crossover design was used to compare two dietary conditions: (1) a FDPIR diet that met DGA and (2) a FDPIR diet that did not meet DGA. Each participant served as their own control and was exposed to both dietary conditions. Repeated-measures ANOVA and *t* tests assessed significance between the two dietary conditions.

**Setting:**

This took place in the Montana State University Nutrition Research Laboratory in the USA.

**Participants:**

Female and male participants (*n* 13) aged 18–55 years from the university and local community.

**Results:**

There were no significant differences in inflammatory response and appetite sensations between the two dietary conditions. Findings indicated that participants ate 14 % more (*P* < 0·01) kcal on a typical FDPIR diet compared with a FDPIR diet that met DGA.

**Conclusions:**

Higher energy intake during a typical FDPIR diet compared with a FDPIR diet that meets DGA may increase risk for obesity and nutrition-related diseases, including type 2 diabetes and other chronic inflammatory conditions.

American Indian and Alaskan Native (AI/AN) communities are disproportionately affected by type 2 diabetes mellitus (T2D) compared with other races and ethnicities in the USA^(1–3)^. According to the Centers for Disease Control and Prevention National Diabetes Statistics Report, AI/AN have the highest rate (15·9 %) of diabetes among adults over the age of 18 years among all races and ethnicities in the USA^(3)^. AI/AN populations have a 50 % higher chance of becoming obese than non-Hispanic whites, making obesity another concern for AI/AN communities^(4)^. Obesity is a risk factor for the development of T2D. Food insecurity is closely linked with obesity and development of T2D^(5,6)^. Household food insecurity is almost 200 % higher in AI/AN households compared with non-AI/AN households^(7,8)^. Supplemental food programmes play a critical role in food accessibility for households with low food security^(9,10)^. The Food Distribution Program on Indian Reservations (FDPIR), also known as Commodities, is the United States Department of Agriculture’s primary supplemental food programme that serves income-eligible AI/AN households on reservations, designated areas around Oklahoma, and through nineteen tribal agencies in Alaska^(11,12)^. Although the FDPIR foods are only meant to be supplemental, almost 40 % of FDPIR recipients on reservations rely on the FDPIR monthly food packages as their sole source of food^(13)^. This is a concern because the average FDPIR monthly food package does not meet Dietary Guidelines for Americans (DGA). On average, the food packages have excessive amounts of low-quality carbohydrate foods, such as refined grains, and lack nutrient-dense carbohydrate sources such as fresh fruits and vegetables^(14)^.

Carbohydrate food quality is measured by nutrient and energy content. Nutrient-dense carbohydrate sources are high in fibre, minerals and vitamin content, all of which have the benefits of prolonging secretion of appetite-regulating hormones, increasing viscosity of intestinal content and slowing gastric emptying^(15–17)^. Low-quality carbohydrate foods have added sugar, are more energy-dense and have high glycaemic indexes, which can cause a quick spike in blood glucose^(15,18)^. Glycaemic index is the measure of a food’s blood glucose-raising ability. Low-quality carbohydrate foods like refined grains can directly and indirectly lead to metabolic disturbances that are important links in the development of T2D and other chronic inflammatory diseases^(18–22)^. For instance, low-quality carbohydrate foods can cause inflammation by increasing oxidative stress, which then stimulates the production of pro-inflammatory cytokines, such as IL-1*β* and IL-6^(18,20)^. IL-1*β* is a central mediator in the cytokine networks, meaning that it is essential in stimulating further production of pro-inflammatory molecules. IL-6 is both anti- and pro-inflammatory cytokine. Both cytokines are commonly used to measure inflammation in humans^(23,24)^.

Indirectly, low-quality carbohydrate food can increase inflammation by elevating visceral adiposity^(19,25)^. Previous research studies have demonstrated that energy-dense, low-quality carbohydrate foods can induce overeating and cause weight gain^(25,26)^. Visceral adipose tissue is elevated in obese individuals and is considered a direct risk for T2D^(19)^. Visceral adipose tissue can accumulate leucocytes, which produce pro-inflammatory cytokines, including IL-1*β* and IL-6^(27,28)^. Elevated pro-inflammatory cytokine production can obstruct adipocyte function by disrupting translocation of insulin signalling for glucose uptake. Inflammation in visceral adipose tissue can also disrupt free fatty acid uptake from the blood, which can lead to elevated free fatty acid in the blood. Consequently, this can cause hepatic insulin resistance and further production of pro-inflammatory molecules^(29)^. To summarise, low-quality carbohydrate food, like the refined grains offered through the FDPIR, have the potential to increase visceral adiposity and the production of pro-inflammatory molecules. This then carries the risk of reducing insulin sensitivity and increasing the risk for developing chronic inflammatory conditions.

The purpose of this study was to compare the effects of a typical FDPIR diet with a FDPIR diet that met DGA on inflammation, appetite and energy intake on a combination of adult AI and non-AI individuals. It was hypothesised that there would be a significant difference in IL-1*β*, appetite and energy intake measurements between the dietary conditions. Learning how FDPIR foods influence inflammation, appetite and energy intake could aid in better understanding how FPDIR impacts the health and well-being of AI/AN communities. Knowledge gained from this study will improve our understanding of the health impacts of FDPIR diets and also enable Indian Tribal Organizations, state agencies and FDPIR policymakers to make decisions that support the health status of the AI/AN communities that rely on FDPIR.

## Experimental methods

### Participants

Three AI and ten non-AI participants (*n* 13), 18–55 years of age (male = 3, female = 10), enrolled in the study. Inclusion criteria included males and females within the ages 18–55 years, waist circumferences ≥94 cm for men and ≥80 cm for women and having an activity level between sedentary or moderate. Exclusion criteria included taking prescribed or over-the-counter anti-inflammatory medication or lipid lowering medication, having an acute or chronic inflammatory disease including diabetes, periodontal disease, kidney problem and heart disease, under or over the age range of 18–55 years, and females who are pregnant. Participants were informed of potential risks and discomforts of the study and signed an informed consent document approved by the Institutional Review Board for protection of human subjects at Montana State University (MSU). Participants were recruited within the MSU campus, the city of Bozeman and surrounding areas using flyers, emails and word of mouth. Flyers were placed around MSU campus and shared via email to all AI/AN MSU students by the MSU American Indian Student Center.

### Research design

**Fig. 1:**
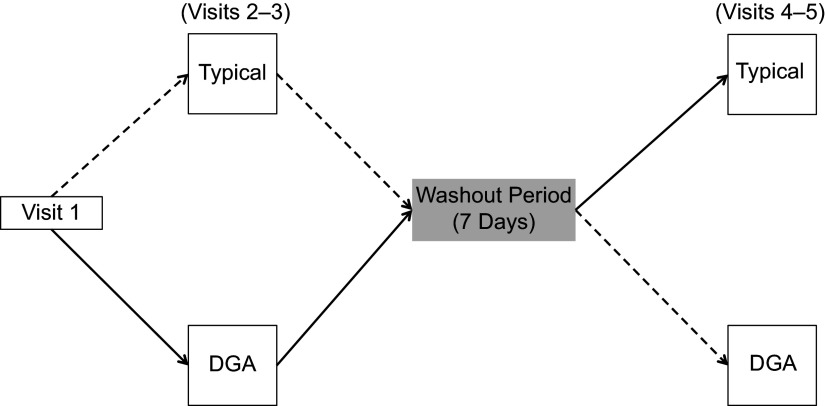
Visit 1 involved reviewing and signing the informed consent document and collection of anthropometric measurements (%fat, %lean, waist circumference, weight and height). ‘Typical’ represents the typical Food Distribution Program on Indian Reservations (FDPIR) dietary condition and ‘DGA’ represents the dietary condition that met Dietary Guidelines for Americans (DGA). Participants were randomly assigned to one of the conditions for visits 2–3, then there was a 7-day washout period before crossing over to the second condition (visits 4–5)

A within-subjects, randomised, crossover design was used to compare two dietary conditions: (1) a FDPIR diet that met DGA (DGA dietary condition) and (2) a FDPIR diet that did not meet DGA (typical dietary condition). Table 1 shows the list of food for each dietary condition. Participants served as their own control and were exposed to both dietary conditions. Using simple randomisation, the participants were randomly assigned to a dietary condition upon completing their initial visit. The order of the two dietary conditions was counterbalanced between participants and separated by at least 7 d to allow variables to return to baseline between diets. Previous studies have observed that a 7-d washout period allows inflammatory cytokines to return to baseline^(30)^. Figure 1 is a diagram of the project’s randomised crossover design. The study took place in the MSU Nutrition Research Laboratory and MSU Herrick Hall Foods Laboratory on the MSU campus. Participants were asked to complete five visits.

**Table 1:** Food list

	FDPIR typical diet	FDPIR DGA diet	
1. 1 % milk	2. Cornflakes	1. Regular oatmeal	2. Wild rice
3. Canned fruit cocktail	4. Saltine crackers	3. 1 % milk	4. Black beans
5. American cheese	6. Macaroni & cheese	5. Fresh grapefruit	6. American cheese
7. Unsweetened applesauce	8. Spam	7. Chicken breast	8. Whole wheat tortilla
9. Spaghetti noodles	10. Spaghetti sauce	9. Romaine lettuce	10. Fresh apples and oranges
		11. Dried fruit and nut trail mix	12. Pork chops
		13. Steamed broccoli	14. Mashed sweet potatoes

FDPIR, Food Distribution Program on Indian Reservations; DGA, Dietary Guidelines for Americans.

### Experimental protocol: baseline assessment

Each test day lasted for a total of 24 h. The initial visit occurred between 08.00 and 09.00 hours, 2–5 d prior to the first test day. This visit took approximately 1 h for each participant and included the following activities: (1) reading and signing the written informed consent document; (2) completing the screening questionnaire; (3) reviewing the appetite and post-condition questionnaires; (4) reviewing the instructions for self-collecting saliva samples and (5) taking anthropometric measurements using a stadiometer (Perspective Enterprises) to measure height, an ergonomic circumference measuring tape (Seca) to measure waist circumference, and a bioelectrical impedance analyzer (Seca) and an air displacement plethysmography (COSMED BodPod) to measure body composition, weight, RMR and estimated total energy expenditure. Baseline saliva samples were collected at 07.00 hours on each test day prior to breakfast to measure pro-inflammatory biomarkers IL-1*β* and IL-6. Baseline appetite ratings for hunger, fullness, satiety, desire to eat and prospective consumption were collected immediately following the collection of each baseline saliva sample.

### Visits 2 through 5

**Fig. 2:**
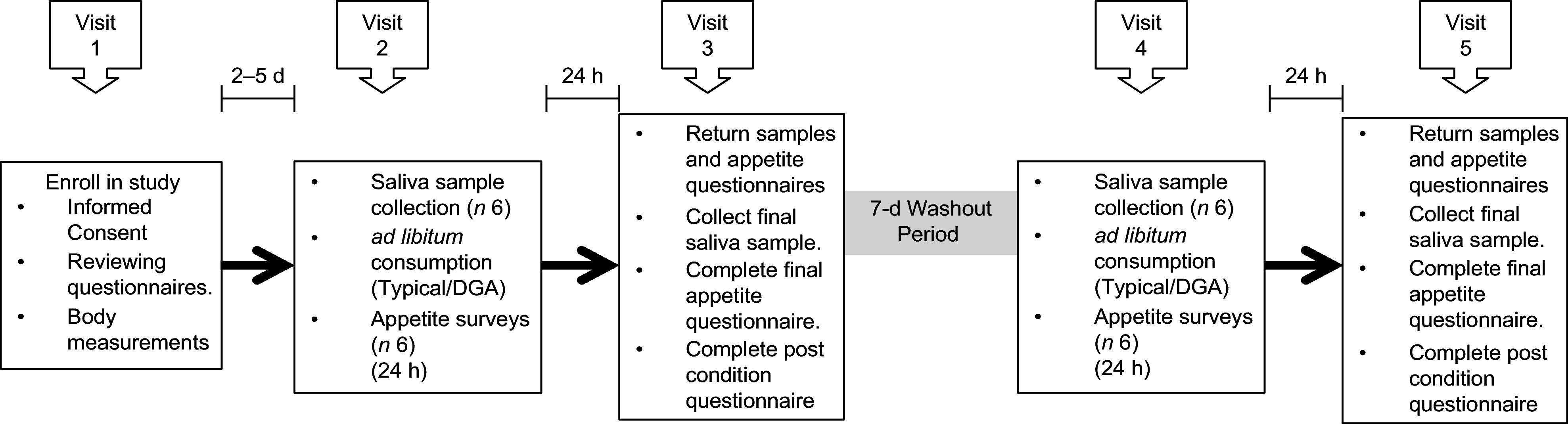
Diagram of the experimental protocol. ‘Typical’ represents the typical Food Distribution Program on Indian Reservations (FDPIR) dietary condition and ‘DGA’ represents the dietary condition that met Dietary Guidelines for Americans (DGA)

Visit 2 occurred 2–5 d after visit 1. Participants were screened to ensure they still met all exclusion and inclusion criteria and were asked to refrain from exercise and/or vigorous activity, consuming alcohol and smoking 24 h prior to and the day of each test day in order to minimise variability in inflammatory concentrations. Participants were also asked to log their diet the day before visit 2 in order to help participants replicate their food choices the day before their next test day (visit 4). Participants were instructed not to consume anything except water in the morning of visits 2 through 5. Visit 2 took approximately 1 h and entailed collecting participants’ baseline saliva sample and appetite ratings, providing breakfast and providing participants with saliva collection kits, appetite questionnaires and food for the remainder of the day. Participants were instructed to eat *ad libitum* and to restrict food consumption during the entirety of each test day to the food that was provided to them. Previous *ad libitum* feeding studies have assessed the effects of one meal on appetite and energy intake^(31,32)^. This study focused on one full day of *ad libitum* feeding for each diet. The following morning (visit 3), participants returned to the MSU Nutrition Research Laboratory to deliver their saliva samples, completed appetite questionnaires and uneaten food. During this visit, participants provided their final saliva sample for the dietary condition and completed an appetite questionnaire and the post-condition questionnaire. At least 7 d after completing the first dietary condition, participants crossed over to the second dietary condition and repeated the procedures of visits 2 and 3. For each dietary condition, participants self-collected six saliva samples and completed six self-reported appetite questionnaires and one post-condition questionnaire (total of twelve samples, twelve appetite questionnaires and two post-condition questionnaires). All five visits took approximately 2–4 weeks. Figure 2 displays a summary of the experimental protocol, and Table 2 displays the experimental protocol and time course for collecting saliva samples and completing appetite questionnaires.

**Table 2:** Experimental protocol and time course

Visit	Time	Activity or procedure
1	08.00 hours	Initial laboratory visit (informed consent, screening questionnaire, fat%, lean%, weight, height and waist circumference)
2	Waking to sleeping	Controlled diet, limited physical activity
	07.00 hours	1st baseline saliva collection and appetite questionnaire
	11.00 hours	2nd saliva collection and appetite questionnaire
	14.00 hours	3rd saliva collection and appetite questionnaire
	17.00 hours	4th saliva collection and appetite questionnaire
	20.00 hours	5th saliva collection and appetite questionnaire
3	07.00 hours	6th final saliva collection and appetite questionnaire
4	Waking to sleeping	Controlled diet, limited physical activity
	07.00 hours	1st baseline saliva collection and appetite questionnaire
	11.00 hours	2nd saliva collection and appetite questionnaire
	14.00 hours	3rd saliva collection and appetite questionnaire
	17.00 hours	4th saliva collection and appetite questionnaire
	20.00 hours	5th saliva collection and appetite questionnaire
5	07.00 hours	6th final saliva collection and appetite questionnaire

### Saliva sample protocol

Salimetrics salivabio oral swab collection instructions were used to educate participants on how to self-collect saliva samples. The collection instructions required all participants to rinse their mouths, place the swab under their tongues and minimise the movement of the swab for 1–2 min or until the swab was saturated^(33)^. For each sample, two swabs were collected to ensure adequate saliva for both IL-1*β* and IL-6 analyses. The collection schedule for this study was based on the inflammatory response of two pilot participants who underwent both dietary conditions and collected ten saliva samples for each dietary condition. Following the analyses of the pilot participants’ saliva samples, six collection times were selected: 07.00, 11.00, 14.00, 17.00, 20.00 and 07.00 hours the following morning. Immediately following the collection of a saliva sample, participants were asked to store them in a freezer (4°C). The following morning (visits 3 and 5), participants brought their saliva samples into the MSU Nutrition Research Laboratory. There, the participants collected a final sample, and then all samples were stored in an −80°C laboratory freezer until they were analysed. A total of six collection tubes and twelve swabs were provided to the participants for each condition. Salivary IL-1*β* and IL-6 were analysed using ELISA assays (Salimeterics) according to the instructions of the manufacturer. Plates were analysed using a µ-Quant plate reader with 450 nm and 620–630 reference filters (Bio-Tek Instruments Inc.). Samples were analysed in duplicates.

### Assessment of hunger and satiety

The Self-Reported Appetite Questionnaire was a validated survey that contains visual analogue scales with words anchored to each end of a 100 mm scale^(34)^. These words expressed the most positive and negative rating for each of these appetite sensations: hunger, satiety, fullness, desire to eat and perspective consumption. For example, for hunger, ‘Not at all’ is anchored on one end of the scale and ‘Extremely (as hungry as I’ve ever been)’ is anchored on the other end of the scale^(34^^)^. The goal of the questionnaire was to determine if there was a difference in appetite scores between the two dietary conditions. The validity of this approach to collecting appetite data has been established^(34)^. During visit 1, the researcher reviewed the appetite questionnaire instructions and the required completion times with each participant. The appetite questionnaires were completed following self-collection of each saliva sample. There were four goals of the post-condition questionnaire: (1) assess participant compliance; (2) gauge the participants’ overall satisfaction; (3) determine whether or not participants felt that they received enough food and (4) learn if the participants would eat the food on a normal day. Post-condition questionnaires contained five questions. Table 3 displays the questions that were included in the post-condition questionnaire. During the initial visit, the post-condition questionnaire was reviewed and the expected time and place in which the participant was expected to complete the questionnaire were discussed.

**Table 3:** Post-diet questionnaire: question list

Type of question	Questions
Likert Scale	1. I would eat the provided food on a normal day.
Likert Scale	2. I had enough food to last me through the day.
Likert Scale	3. Overall, I am satisfied with the food that was provided.
Yes/No, explain	4. Did you eat or drink anything outside of the food and water that was provided? If yes, please list the food/beverage items that you consumed outside of the provided foods.
Yes/No, explain	5. Did you not eat a food item that was provided to you?

### Diet composition

Food for both dietary conditions was selected from the FDPIR food list from Exhibit O FNS Handbook 501^(35)^. The typical dietary condition was constructed to have comparable healthy eating index (HEI) scores to both the US average adult HEI scores, and the HEI scores found by an assessment completed by Byker Shanks *et al*.^(14)^, which assessed the nutritional quality of food packages offered in the FDPIR using HEI 2010. The average FDPIR monthly food package scored an average of 66·38 ± 11·60. The national average HEI score for adults 18–64 years of age between 2015 and 2016 was 58·3^(36)^.

In the present study, the typical dietary condition consisted of 1 % milk, cornflakes, canned fruit cocktail, saltine crackers, American cheese, macaroni and cheese, unsweetened applesauce, spam, spaghetti noodles and spaghetti sauce with ground beef. The DGA dietary condition was constructed to score a minimum HEI score of eighty out of 100. The DGA dietary condition consisted of regular oatmeal, 1 % milk, fresh grapefruit, chicken wraps (chicken breast, romaine lettuce, wild rice, black beans, American cheese and whole wheat tortilla), fresh assorted fruit (apples and oranges), a fruit and nut trail mix, pork chops, steamed broccoli and mashed sweet potatoes. The National Cancer Institute Automated Self-Administered 24 h Dietary Recall System (ASA24) was used in developing each dietary condition. The ASA24 report provided essential nutrient information to calculate HEI scores. HEI scores were calculated using the simple HEI scoring method. First, the ratio of the dietary constituent to energy was constructed and scored according to the scoring standards. The component scores were summed to calculate the total score^(37)^. The constructed typical dietary condition had a HEI score of 53·8, and the constructed DGA dietary condition had a HEI score of 89·5.

All food was cooked, stored and served in accordance with ServSafe Montana standard operating procedures. ServSafe is a food and beverage safety training and certificate programme administered by the National Restaurant Association. Researchers handling the food for the study were ServSafe certified. Breakfast was served to participants in the MSU Herrick Hall Food Laboratory. After breakfast, participants were provided with food for the remainder of the day according to their estimated total energy expenditure, which was estimated by the air displacement plethysmography machine. Kilocalories were calculated by weighing the food in grams and then multiplying by the number of kcal per gram for each food. The kcal per gram for each food item were determined using the food item’s nutrition labels and myfitnesspal.com. With the exception of water, participants were asked to restrict consumption to the provided food. The participants were allowed to eat *ad libitum*. Uneaten food was returned to the laboratory and weighed the following morning to determine total energy consumption.

### Statistical analysis

Data were analysed using the Statistical Package for the Social Sciences software, and Microsoft Excel. Descriptive statistics were used to describe participant demographics and anthropometric measurements. A repeated-measures ANOVA was conducted to evaluate the null hypothesis that there was no inflammatory response difference between the two dietary conditions, and it was also used to examine the effect of each dietary condition on appetite sensations. AUC was calculated for IL-1*β* concentrations for each participant, and a paired *t* test was conducted to determine if there were significant differences between each dietary condition. A paired *t* test was also used to compare daily energy intakes and mean appetite ratings for each dietary condition. Criterion for statistical significance was *P* < 0·05.

## Results

Three AI and ten non-AI participants, 18–55 years of age (male = 3, female = 10), enrolled in the study. Participants ranged from 19 to 53 years of age, with the mean age of 32 ± 10·37 years of age. The mean BMI for the group was 28·69 ± 5·0 kg/m^2^. None was smokers, pregnant or had a history of diabetes or any other inflammatory illness. Participant anthropometric and demographic characteristics are shown in Table 4.

**Table 4:** Participant characteristics

	AI females (*n* 3)		Non-AI females (*n* 7)			Non-AI males (*n* 3)	
Variables	Mean	sd	Mean	sd	AI males (*n* 0)	Mean	sd
Age (years)	33·3	16·82	26·7	8·71		37·7	10·02
Height (cm)	161·25	11·11	145·21	8·69		182·1	12·52
Body mass (kg)	89	18·36	66·45	13·73		104·6	13·76
Body fat (%)	40·4	11·98	30·58	6·42		34·2	3·54
Waist circumference (cm)	110	14·59	74·68	3·71		107·5	11·46
BMI (kg/m^2^)	33·46	9·38	23·75	2·47		27·8	4·91

AI, American Indian.

### Inflammatory biomarkers

**Fig. 3:**
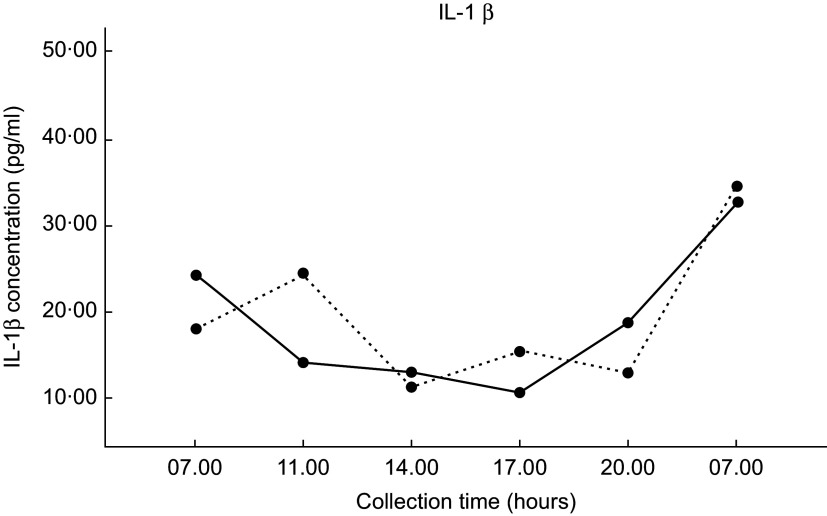
Values are the mean IL-1*β* concentrations (±1 se) for each of the six collection times. DGA, solid line (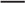); TYPICAL, dashed line (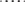). ‘Typical’ represents the typical Food Distribution Program on Indian Reservations (FDPIR) dietary condition and ‘DGA’ represents the dietary condition that met Dietary Guidelines for Americans (DGA)

Mean IL-1*β* concentrations for each dietary condition are shown in Fig. 3. The salivary IL-6 concentration did not reach detection level in the majority of participant saliva samples; thus, IL-6 cytokine data were excluded. Salivary IL-1*β* cytokines were detected in all participants’ saliva samples. There were no significant differences in IL-1*β* concentrations between the two dietary conditions found by repeated-measures ANOVA (*P* = 0·591). There were also no significant differences in AUC calculations between the two dietary conditions found by paired *t* test (*P* = 0·358).

### Perception of appetite

**Fig. 4:**
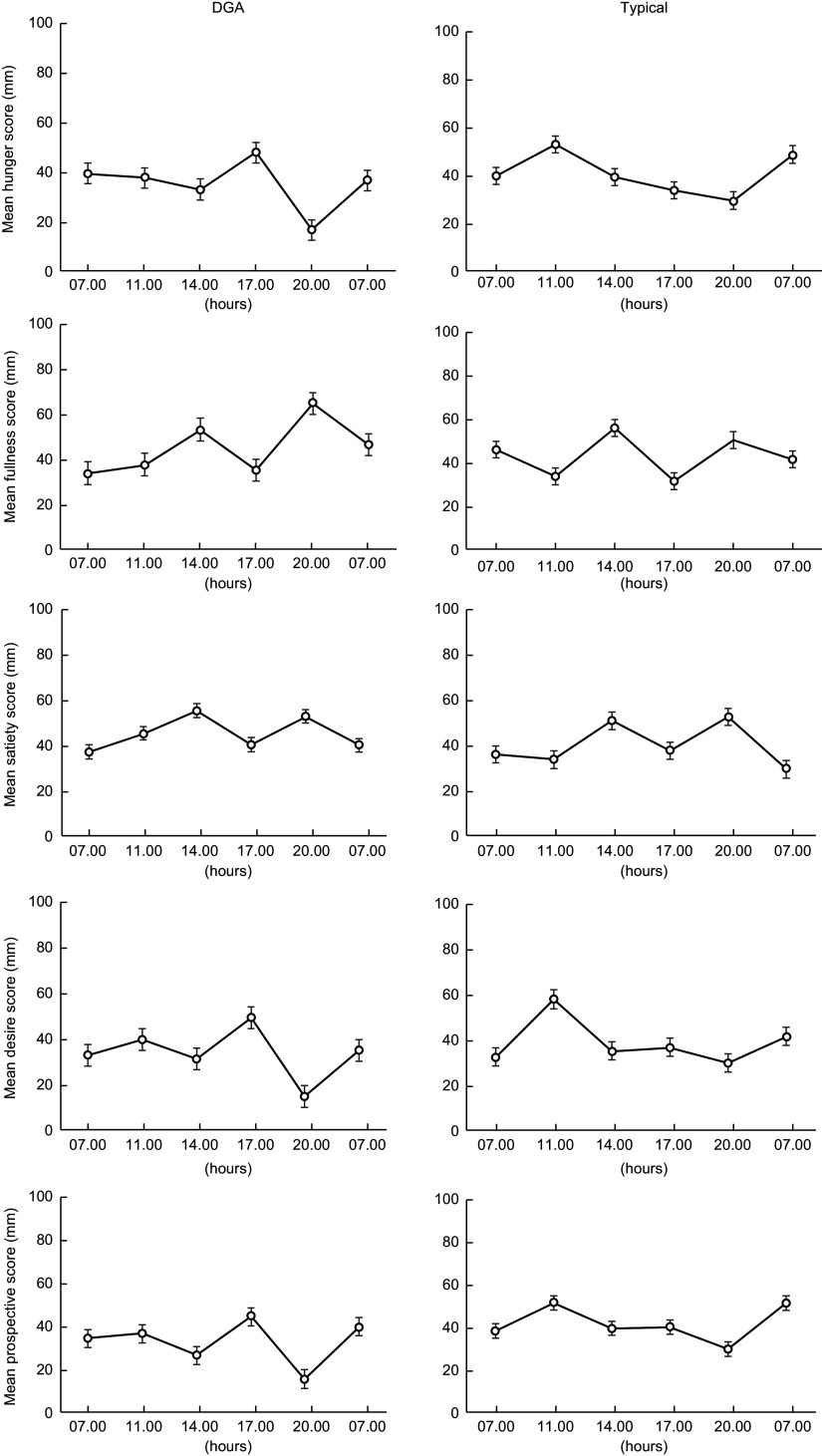
Values are the mean participant appetite scores for each sensation. The six survey completion times were completed over 24 h (07.00, 11.00, 14.00, 17.00, 20.00 and 07.00 hours). The first value in each graph is the mean baseline score

**Fig. 5:**
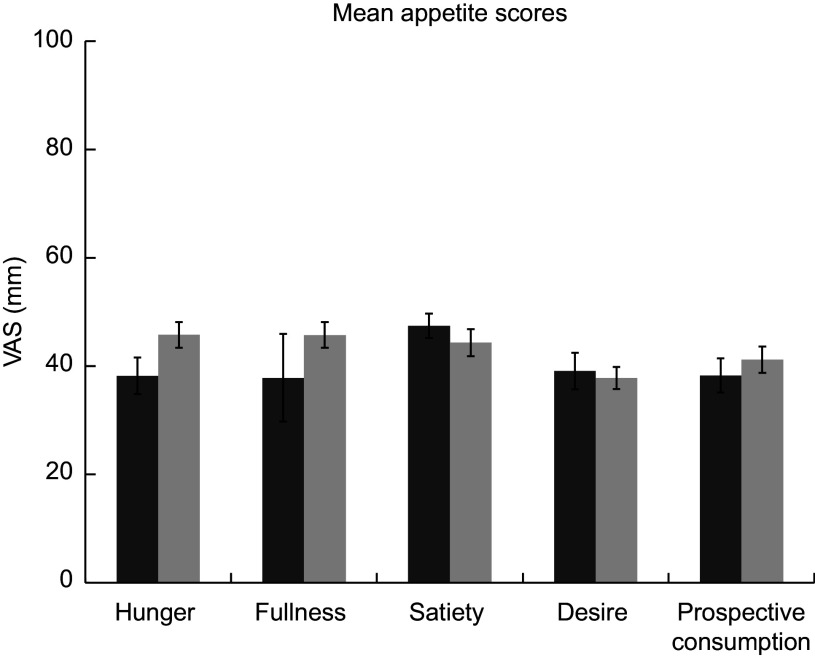
Total mean appetite scores for both conditions. DGA, dark bars (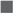); TYPICAL, light bars (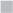). ‘Typical’ represents the typical Food Distribution Program on Indian Reservations (FDPIR) dietary condition and ‘DGA’ represents the dietary condition that met Dietary Guidelines for Americans (DGA)

The response curves for each appetite sensation (hunger, satiety, fullness, desire to eat and perspective consumption) are presented in Fig. 4. The appetite profiles were similar after each dietary condition. Appetite scores were not significantly different between the two dietary conditions in all sensations found by paired *t* test (*P* < 0·05). Figure 5 presents the total mean appetite scores for each condition. The mean appetite sensation scores for each collection time were not significantly different between the two dietary conditions according to repeated-measures ANOVA (*P* < 0·05).

### Total daily energy consumption

Table 5 presents the mean and median daily energy intakes ± se for the two dietary conditions. Paired *t* test showed significant differences in absolute daily energy (kcal/d) between the two dietary conditions (*P* = 0·006). On average, participants on the typical FPDIR condition consumed 14·08 % more kcal than when on the FDPIR DGA condition. The post-condition questionnaires revealed that three participants consumed food that was not provided to them during the DGA condition, and six participants during the typical condition. The estimated kcal from those food items were added to the participant’s total daily energy consumption.

**Table 5:** Total daily energy intakes

Variables	Mean (*n* 13)	se	Median (*n* 13)
DGA (daily energy intake) (kcal/d)†	1979·31	129·75	1860
Typical (daily energy intake) (kcal/d)†	2303·73**	140·58	2285

DGA, Dietary Guidelines for Americans.

** *P* < 0·01 compared with DGA condition.

† To convert kcal to kJ, multiply it by 4·184.

### Satisfaction with typical *v.* DGA diets

**Fig. 6:**
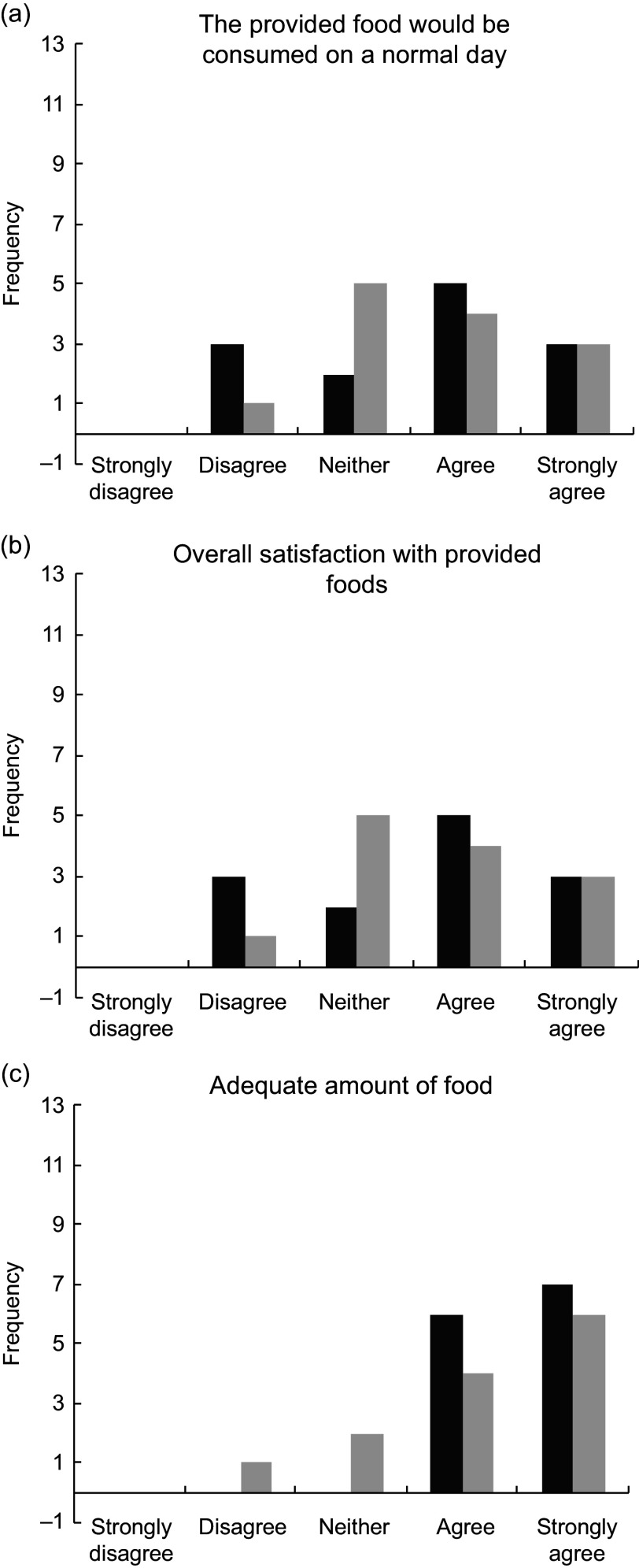
Graphs display the frequency of the participant responses to the post-condition questionnaire Likert scale questions. DGA, dark bars (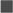); Typical, light bars (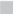). There are no significant differences between the responses for each question found by paired *t* test (*P* < 0· 05)

Participant responses to the post-condition questionnaire Likert scale (1–5) questions are presented in Fig. 6. The participants’ average response for whether or not they would eat the food from the typical dietary condition on a normal day was 2·6 (between disagree and neither agree nor disagree). The average response for whether or not they would eat the food from the DGA dietary condition on a normal day was 3·0 (neither agree nor disagree). The participants’ average response to describe their overall satisfaction with the food from the DGA dietary condition was 3·6 (between neither agree nor disagree and agree) and was 3·69 (same) for the food from the typical dietary condition. For the last Likert question, the participants agreed or strongly agreed (4 or 5) that they had enough food for the duration of both test days (DGA = 4·5; typical = 4·2). The responses for each of the three questions were not significantly different between the two dietary conditions found by paired *t* test (*P* < 0·05).

## Discussion

Understanding how food from the FDPIR influences inflammatory response, appetite and energy intake provides a broader view of how the FDPIR influences risk for nutrition-related diseases among AI communities, including obesity and T2D. Furthermore, understanding how aligning with the DGA is important with respect to nutrition-related disease prevention and management among AI communities. Awareness about these linkages will enable Indian Tribal Organizations, related state agencies and FDPIR policymakers to better support the health status of AI/AN communities that rely on FDPIR food.

The main finding of the study was that average daily energy intake was significantly higher after the typical condition compared with the DGA condition. On average, participants consumed over 14 % more kcal upon completing the typical condition. Palatability, physical activity, energy availability and nutrient density have been identified as factors that can affect energy intake. To account for palatability differences, the post-condition questionnaire measured how satisfied participants were with the food. Satisfaction ratings were similar between the two dietary conditions. Similar satisfaction ratings for both dietary conditions reduced the likelihood that the energy intake differences were simply due to participants finding one dietary condition more palatable than the other.

To control for physical activity, participants were instructed to avoid moderate to vigorous physical activities the day before and the day of each test day. Participants’ estimated total energy expenditure was used to determine the amount of energy provided to them during each dietary condition. Balancing available kcal for each dietary condition reduced the chance of energy intake differences due to dissimilarities in energy availability. Adequate energy was also confirmed by post-condition questionnaire responses. Participants agreed or highly agreed that they had enough food to last them throughout each test day. Hence, inadequate availability of kcal was eliminated as a potential explanation for daily energy intake differences.

Differences in energy intake between the dietary conditions may have been due to energy density differences. The typical dietary condition contained more low-quality carbohydrate foods and fell short on fresh fruit and vegetable options, which is similar to an average FDPIR monthly food package^(14)^. The DGA dietary condition had more fresh fruits and vegetables and limited added sugar. For example, a half-cup serving of canned fruit cocktail has 60 energies (15 g carbohydrates (1 g fibre and 12 g sugar) and 1 g protein); and a half-cup serving of a fresh cut apple has 29 energies (8 g carbohydrates (1·3 g fibre and 6 g sugar) and 0·2 g protein). Thus, if one were to consume a half-cup of canned fruit cocktail, they would consume a little over twice the amount of energies that they would if they instead ate a half-cup of a fresh cut apple.

Previous research revealed that individuals are not sensitive to energy density and will eat the same volume of food when presented with dietary conditions that vary in energy density, which results in significant energy intake differences^(38)^. One study found that appetite (hunger and fullness) remained unchanged during *ad libitum* consumptions of meals that varied in energy density^(39)^. This is supported by the current study’s second finding which was that appetite sensation ratings were not significantly different between the two dietary conditions. The appetite sensation ratings remained the same between dietary conditions, despite differences in the energy density of the foods provided for each condition. We determined that appetite sensation ratings were similar between the two dietary conditions because the participants consumed enough food during each dietary condition to satisfy their appetites (hunger, fullness, satiety, desire to eat and prospective consumption). Consequently, the total daily energy intake was significantly higher post-typical dietary condition compared with post-DGA dietary condition.

In summary, the combination of higher energy intake after the typical dietary condition along with similar appetite sensations between both dietary conditions may be an indication that the participants adjusted their energy intake to satisfy feelings of hunger and satiety. There are not many studies on the effects of various foods on energy intake and appetite in AI populations. Further research investigating nutrient composition of each FDPIR diet and energy intake is needed in AI populations.

It was hypothesised that there would be a difference in inflammatory response between the typical dietary condition and the DGA dietary condition. Based on prior research, it was expected that there would be a larger inflammatory response after the typical dietary condition, which provided a greater amount of low-quality carbohydrate foods than the dietary condition that met DGA standards^(18–20)^. Low-quality carbohydrate foods can directly and indirectly cause inflammation^(18,25)^. Excess consumption of low-quality carbohydrate foods is associated with increased visceral adiposity, which is an important link to suppressed peripheral and hepatic insulin sensitivity, and elevated pro-inflammatory cytokine production^(29,40)^.

Acutely, low-quality carbohydrates elevate blood glucose, which in turn increase production of reactive oxygen species, or oxidative stress, by the mitochondria of cells. Consequently, reactive oxygen species stimulates the production of inflammatory cytokines, including IL-1*β* ^(18,20)^. A previous study found that IL-1*β* levels are augmented 24 h after a high carbohydrate meal^(41)^, which helped inform our study design test day length. Our current experiment however did not confirm previous studies that have reported increased inflammation following consumption of low-quality carbohydrate food. One possibility for this is that the previous studies that show an increase in inflammation from carbohydrate ingestion used serum cytokines to determine inflammatory response, while we used salivary cytokines. Kesseler *et al*.^(42)^ showed that timing of carbohydrates and fat ingestion has minimal effect on inflammatory biomarkers in saliva, which could have implications for our chosen study design. Not detecting a significant difference in inflammatory response between the two conditions may have also been due to a small sample size. Increasing the sample size may provide a better estimation of the mean measurements, thus allowing for more accurate comparisons of inflammatory response between the two dietary conditions. Furthermore, IL-6 measurements not reaching the detection limit may have been due to salivary flow rate, which has been found to affect concentration of biomarkers in saliva^(42,43)^. An assessment of saliva rate was not conducted because the participants self-collected their saliva samples at home. Other possible explanations include lack of precision in saliva sample collection and storage^(43)^. Although participants were provided detailed instructions, we could not ensure proper at-home saliva collection and storage of all samples.

There were limitations to the study design. First, the small sample size was a limitation. Second, the food items were purchased from a local grocery store, not from a FDPIR distribution site. Therefore, nutrient composition was similar, but not exactly the same as FDPIR foods. Future studies could include food directly from the FDPIR. Third, although participants agreed to adhere to several diet and physical activity restrictions 24 h prior to and the day of each dietary condition, the post-condition questionnaire revealed that multiple participants ate food outside of the food that was provided to them for each dietary condition. The items were mostly small snack type foods. The estimated kcal from the added food were included in the total daily energy intake measurements. The effects of the added food on each variable were unknown and therefore are a limitation of the study. Potentially, the added foods could have affected inflammation levels within the participants who consumed them^(44,45)^. It was beyond the resources of the study to monitor the participants outside of the laboratory to ensure participant adherence.

Fourth, there were no AN participants and only three AI participants in the study. The participants were primarily non-AI, which was a limitation of the study because FDPIR recipients are mostly AI/AN who live on a reservation or in designated areas in Oklahoma and Alaska. Thus, the majority of the participants lacked knowledge of and exposure to FDPIR foods prior to participating in the study. Future recruitment efforts could be improved by partnering with local AI/AN programmes and Tribes. Fifth, women who were postpartum were not excluded from the study. Research shows the potential for elevated inflammation during the postpartum period^(46)^. Additionally, recent vaccinations were not screened for, which could have affected inflammation levels^(47)^. It was unknown whether any of the participants were postpartum and/or had recent vaccinations. Sixth, the effects of the foods that the participants consumed prior to each test day are unknown.

Lastly, available healthy food options vary among FDPIR distribution sites. The dietary conditions for this study were constructed using Exhibit O of the FNS Handbook 501, which contains all FDPIR food items. At the state and tribal level, FDPIR distribution site managers have the authority to decide which FDPIR food items to stock^(48)^. For example, one FDPIR site could offer all food items that are available through FDPIR, and another could opt out of ordering healthier foods, such as fresh produce. In that case, selecting foods that meet DGA standards may be limited. Therefore, the dietary conditions constructed for this study, particularly the dietary condition that met DGA standards, may not be realistic for FDPIR recipients that are limited by the lack of healthy food options at their FDPIR distribution site. Providing nutrition education to FDPIR distribution site managers and staff could aid in ensuring that all FDPIR fresh produce options are available at every site.

Despite the limitations to the study design, our finding of 14 % higher energetic intake in a typical FDPIR diet *v*. a FDPIR diet that met DGA standards suggests that the FDPIR programme has the potential to promote weight gain, which may increase risk for obesity and nutrition-related diseases among AI/AN communities, including T2D. The results of the study indicate that it may be beneficial for the health status of many AI/AN communities for FDPIR distribution sites to align with DGA by providing an adequate amount of high-quality carbohydrate options, and by providing education on healthy diets to FDPIR recipients.

In conclusion, IL-1*β* measurements and appetite sensation ratings were not significantly different between dietary conditions. Daily energy intake was significantly higher after the typical dietary condition compared with the dietary condition that met DGA standards. This is the first study that we know that has demonstrated the effects of both a typical FDPIR diet and a FDPIR diet that met DGA standards on inflammation, energy intake and appetite ratings. With respect to preventing and controlling T2D and obesity in AI/AN communities, these are important findings because a typical FDPIR diet or a diet that has similar nutrient content may promote a positive energy balance, which can lead to weight gain and increased risk for developing or progressing nutrition-related diseases including T2D. Conversely, a FDPIR diet that aligns with DGA may reduce the risk for developing nutrition-related diseases by limiting FDPIR foods with added sugar and thus potentially preventing excess energy intake. This study exposed the need for further research on the long-term effects of food from the FDPIR on metabolic health. Expanding our knowledge on how FDPIR food affects inflammation, appetite and energy intake is important for supporting chronic disease prevention and management in AI/AN communities, and enabling policymakers to make decisions regarding the FDPIR that supports the health status of AI/AN populations.
